# Evaluating the effectiveness of behavioural nudges in reducing energy consumption in student accommodation: a quasi-experimental approach

**DOI:** 10.14324/111.444/ucloe.3412

**Published:** 2025-09-04

**Authors:** Youjing Chen, Lorenzo Lotti

**Affiliations:** 1 Institute for Sustainable Resources, The Bartlett School of Environment, Energy and Resources, University College London, UK

**Keywords:** consumption, energy, nudge, behavioural economics

## Abstract

This paper investigates the application of nudge theory to reduce utility consumption within student accommodation, specifically focusing on the effectiveness of informational and competition-based nudges. With the pressing challenge of climate change and the significant contribution of the building sector to global energy use, finding innovative, cost-effective strategies to promote sustainable behaviour is critical. This study employs a quasi-experimental design across six buildings divided into four groups: Control, Information-only, Competition Without Prizes and Competition With Prizes. The research aims to explore the differential effects of informational feedback and competition, with and without prizes, on energy consumption. The study utilises a longitudinal approach, examining energy usage across multiple years to control for external factors such as occupancy fluctuations and seasonal effects. Results reveal that the informational nudge, contrary to expectations, increased energy consumption in certain accommodation, possibly due to rebound effects or moral licensing. Meanwhile, the competition without prizes nudge effectively reduced energy usage, highlighting the power of intrinsic motivation and social comparison. However, the competition with prizes nudge showed no significant effect, suggesting that extrinsic rewards might undermine the intrinsic motivation to save energy. This research contributes to the growing body of literature on behavioural change interventions in residential settings, particularly within transient and dense environments such as student accommodation. The findings underscore the need for nuanced, well-designed nudges that account for behavioural dynamics and suggest that low-cost strategies which utilise intrinsic motivators may be more effective than those that provide extrinsic rewards in fostering sustainable habits in student accommodation. Furthermore, the study highlights the importance of robust communication strategies to enhance the efficacy of behavioural interventions in reducing energy consumption.

## Introduction

Climate change presents a significant global challenge, with energy consumption being a primary contributor to greenhouse gas emissions. The building sector accounts for approximately 40% of total energy consumption, emphasising the need for energy-efficiency interventions [1,2]. While technological advancements have improved building efficiency, discrepancies between expected and actual resource use highlight the importance of behavioural interventions. Behavioural science, particularly nudge theory, offers cost-effective strategies to encourage energy-saving behaviours without major infrastructural changes [3].

Student accommodation present a unique opportunity for implementing behavioural interventions. The habit discontinuity hypothesis [4] suggests that individuals experiencing life transitions, such as moving into student housing, are more receptive to behavioural changes. However, studies indicate that students often exhibit high energy consumption due to a lack of awareness or motivation [5]. Given that UK universities accounted for 1.4 million tonnes of CO_2_ emissions in 2021–2022 [6], effective strategies for reducing student energy use are crucial. Interventions in student accommodation not only have immediate benefits in terms of reduced energy consumption but also serve an educational role. By engaging students in sustainability practices during their stay in these accommodation, there is a significant potential for instilling long-lasting habits that students carry into their future homes and workplaces. This educational aspect extends the impact of energy-saving measures far beyond the immediate environment, contributing to broader societal changes towards sustainability [7]. From an economic perspective, reducing energy consumption in student accommodation can lead to significant cost savings for both the institutions that manage these properties and the students who live in them. Moreover, studies have shown that focusing on sustainability will increase students’ satisfaction [8,9]. Hence, apart from social benefits, it is also in student accommodation’s economic interest to implement sustainable initiatives.

Nudge-based interventions have demonstrated effectiveness in reducing energy consumption. Informational nudges, such as feedback on energy use, have successfully influenced behavioural change [10,11]. Despite some studies demonstrating the effectiveness of the competitions nudge in reducing energy consumption in student accommodation, there remains a gap in understanding the differential effects of providing information alone versus through competition, as all current studies have provided information for participants as a default setting of competition. Moreover, to the best of the authors’ knowledge, no studies before have investigated the effect of a prize versus no prize competition in a student accommodation setting.

This study aims to fill these gaps by evaluating the effectiveness of different nudging strategies in student accommodation. A quasi-experimental design will compare four groups: one receiving no intervention, one receiving informational nudges, one engaged in competition without prizes and one engaged in competition with prizes. The findings will contribute to understanding the relative impact of informational versus competition-based nudges and the role of incentives in energy conservation.

## Literature review

### Nudge theory

At the core of the nudge theory is the recognition that human beings often make decisions that are not in their best interests due to cognitive biases and heuristics. Kahneman and Tversky’s [12] prospect theory provided early insights into these decision-making anomalies, laying a theoretical foundation for nudging by illustrating how people value gains and losses differently. Nudge theory builds on these insights, proposing strategies that align with natural human tendencies and cognitive biases to promote better decision-making.

First popularised by Richard H. Thaler and Cass R. Sunstein, nudge theory posits that by altering the way choices are presented, individuals’ behaviours can be steered without forbidding any options or significantly changing their economic incentives. A key philosophical and ethical foundation of nudge theory is libertarian paternalism. This concept proposes that it is both possible and legitimate for private and public institutions to affect behaviour while also respecting freedom of choice, as nudges steer people in particular directions but do not restrict their liberty to choose otherwise [3,13]. This aspect addresses the ethical concerns about manipulation, which makes nudge particularly appealing to policymakers and large organisations. Sunstein and Reisch [14] extend this principle to the environmental domain, arguing that default-based and feedback nudges – such as automatically enrolling households into green energy programmes or providing consumption benchmarks – can promote large-scale environmental protection efforts while maintaining individual autonomy. Their work emphasises the importance of designing interventions that are both ethically sound and behaviourally effective, making them particularly suitable for addressing sustainability challenges such as energy use in communal settings.

Nudge interventions have been applied across various domains, including health [3,15], finance [16] and the environment [17,18]. In the context of environmental sustainability, nudges have been utilised to reduce energy consumption, enhance recycling rates, and promote water conservation [19]. For instance, simple nudges such as providing feedback on energy use or modifying the default settings on thermostats have demonstrated significant reductions in energy consumption [10]. These interventions work by making sustainable choices easier, more visible and more convenient. Similarly, a study by Ferraro et al. [20] showed that simple informational nudges that inform households about their water usage relative to neighbours led to a significant reduction in water use. Moreover, a meta-analysis by Schubert [19] found that nudges are particularly effective in changing routine behaviours that have environmental impacts, such as energy use and transportation habits. Another well-documented applications of nudge theory in energy conservation is by Schultz et al. [17], who demonstrated the effectiveness of social norms as a nudge in reducing household energy consumption. The researchers provided households with information about their energy usage relative to their neighbours, alongside either a positive or negative emoticon to indicate whether their consumption was below or above average. This intervention, known as the ‘social norm nudge’, resulted in significant energy savings, particularly for households that were consuming more than the average. More recently, the eye nudge implemented by Lorenzo et al. [21] in a large UK university campus shows that information and visual nudges are cost-effective tools to significantly improve waste sorting behaviour.

Thus, when the need for effective and sustainable energy consumption strategies becomes increasingly critical, nudge theory offers a promising avenue. By leveraging behavioural insights to design subtle yet effective interventions, nudge theory can play a pivotal role in shaping sustainable behaviours. The growing body of research supporting the efficacy of environmental nudges points towards their potential to contribute significantly to sustainability efforts globally, aligning individual actions with broader environmental goals.

There are different types of nudges building on different behaviour economic theories, such as default nudges, which change the default settings or pre-selected options, as people often stick with default choices due to inertia [22]; simplification and framing nudges, which simplify information and how options are presented to affect choices [23]; and social norms and feedback nudges, which involve informing individuals about both the behaviours of themselves and others to motivate them to conform to perceived norms [24]. Specifically, this study employs information and competition-based nudges.

### Informational nudges

Informational nudges play a pivotal role in behavioural interventions by subtly influencing decision-making and promoting positive actions. These nudges operate by providing individuals with specific, timely and contextually relevant information that can prompt them to change their behaviour. The concept of bounded rationality, introduced by Simon [25], is fundamental to understanding why informational nudges are necessary. Bounded rationality suggests that individuals do not always make perfectly rational decisions due to cognitive limitations, lack of information or time constraints. Instead, people use heuristics or rules of thumb to make decisions, which can sometimes lead to sub-optimal outcomes. Informational nudges aim to address these limitations by simplifying the decision-making process and providing the crucial information needed to make better choices. By presenting information in a clear, accessible and actionable way, informational nudges help individuals overcome the barriers posed by bounded rationality [3]. Moreover, feedback theory, as part of cybernetics, suggests that providing people with information about their actions leads to adjustments in future behaviour to achieve desired outcomes. Thus, one of the most effective forms of informational nudges involves the use of feedback mechanisms. Feedback provides individuals with real-time or near-real-time information about their behaviour, allowing them to adjust their actions accordingly. In the context of energy consumption, studies have found that people that receive feedback on their energy use tend to reduce their consumption more effectively than those that do not [26–28]. For instance, a study by Darby [11] found that providing households with real-time energy consumption feedback led to reductions of up to 10% in energy usage. Similarly, Gans et al. [29] conducted a natural experiment in Northern Ireland and found that smart meter feedback systems led to statistically significant reductions in residential electricity consumption, demonstrating the value of real-time, easy-to-understand feedback in shaping everyday energy decisions. These suggest that the mere act of making energy consumption visible can encourage consumers to adopt more energy-efficient behaviours. This phenomenon supports the idea that when individuals are made aware of the immediate consequences of their actions, they are more likely to alter their behaviour to achieve better outcomes.

The application of informational nudges in energy conservation takes various forms, ranging from simple feedback mechanisms mentioned above to more complex interventions that incorporate comparisons. For instance, Fischer [26] reviewed various feedback mechanisms and found that feedback comparing present energy consumption to past consumption was particularly effective. The study demonstrated that such feedback led to energy savings of 5–12%, depending on the frequency and format of the information provided. Thus, this study would use an information nudge which involves biweekly feedback mechanisms that inform individuals about their energy usage every two weeks, coupled with comparison to their past usage, intending to guide them towards more energy-efficient practices.

### Competition nudges

Competition-based nudges build on the human tendency for social comparison and competition. Social comparison theory [30] suggest that individuals constantly compare themselves to others in order to evaluate their own behaviour, abilities and achievements. The effectiveness of such competitions is well-supported by literature, indicating that they can effectively mobilise groups towards common goals, enhance engagement and foster a sense of community and collective effort [31]. Studies have suggested that in environments where social identity and group belonging are strong motivators, such as student accommodation, the social norms and the desire to conform to group behaviours can be a very strong factor in driving people towards energy-saving behaviours [32,33]. Past research implementing competitions in student accommodation have consistently demonstrated comparatively large savings. Peterson et al. [34] conducted a classic study in Oberlin that yielded a 30% savings. More recent campaigns in British Columbia [35] and London [36] have also shown savings of 16 to 20%, and a more modest 6.4% in California [37]. Additionally, with over 55,000 participants from five countries, the SAVES programme is currently the largest energy-saving competition globally. It has resulted in an average of 8% electricity savings in participating university dorms [38].

All of the current research, however, give students the data on energy use alongside the competition by default to the best of the authors’ knowledge. They have not differentiated the impact between informational and competition nudges, which means the reduction in energy consumption levels could be achieved by information provision instead of competition. To address this gap, this study would differentiate the effect between information nudge and competition nudges by applying these two different nudges to different treatment groups.

As discussed above, competitions can significantly motivate individuals and groups to change their behaviour by tapping into the natural human tendencies toward rivalry and achievement. However, the dynamics of these competitions can vary significantly depending on whether they include material incentives. At the heart of this distinction lies the difference between extrinsic and intrinsic motivation. According to Deci and Ryan’s [39] self-determination theory, intrinsic motivation refers to engaging in particular behaviour because it is inherently enjoyable or satisfying, whereas extrinsic motivation involves performing an action to earn external rewards or avoid punishment. In competitive contexts, people are motivated to outperform their peers or group members, even when no direct reward is offered. This competitive drive is linked to status-seeking, self-esteem and personal identity. In this context, a competition without prizes nudge can be seen as a non-monetary incentive, where the goal is to leverage people’s natural tendencies toward social comparison and self-enhancement to influence behaviour. These nudges create an environment where individuals feel motivated to improve performance because they seek social recognition, status or personal satisfaction rather than material gain. As individuals become more aware of their ability to perform well relative to others, they gain confidence in their ability to sustain energy-saving behaviours. Thus, in a competition without prizes nudge, the satisfaction of outperforming others and the internal recognition of one’s success are sufficient to sustain engagement in energy-saving efforts.

The introduction of prizes in competition, as a form of extrinsic motivation, has been utilised in various behavioural interventions to enhance participation and engagement by offering tangible rewards. Gneezy et al. [40] articulate that extrinsic rewards are particularly effective when the desired behaviour or task is perceived as uninteresting or unrewarding without additional incentives. In the context of energy conservation, offering prizes could raise the stakes, drawing greater participation by appealing to both competitive instincts and the lure of the reward. This method can quickly elevate awareness and prompt behavioural changes that might otherwise require more time to instigate through intrinsic motivators alone. However, the crowding out theory introduced by Frey and Oberholzer-Gee [41] states that sometimes price incentives could also crowd out the effect of intrinsic motivation. Thus, it is important to differentiate the two effects and find out whether prize incentives would negatively affect the effect of intrinsic motivation in driving students towards more sustainable behaviours in student accommodation. Moreover, by not including a prize in the competition it could reduce the organisation’s financial cost in reducing its tenants’ energy usage, because in real-world applications not all behavioural interventions will have the resources to offer prizes or external rewards. However, to the best of the authors’ knowledge, currently all studies which implemented competition in student accommodation settings include a prize for the winner. By including a group that competes without prizes, this research can provide insights into the effectiveness of low-cost or no-cost interventions, which are often more feasible for widespread implementation. This comparison allows policymakers and practitioners to make more informed decisions about which types of interventions are likely to be most effective in a student accommodation setting. This also covers the gap that no prior studies have differentiated the competition setting between with a prize and without a prize when doing research in student accommodation.

Overall, the three main hypotheses of this study were:

Hypothesis 1: Providing students with informational nudges will lead to a significant reduction in their energy consumption compared to a no-nudge control group.

Hypothesis 2: Students exposed to a competition-based nudge (with information provided) will significantly reduce their energy usage more than students who receive only informational nudges, indicating that the competitive approach has a distinct effect on energy reduction apart from information provision.

Hypothesis 3: An energy-saving competition that includes prize incentives will drive greater reductions in energy consumption than a competition without prizes.

## Method

### Research design

**Figure 1:**
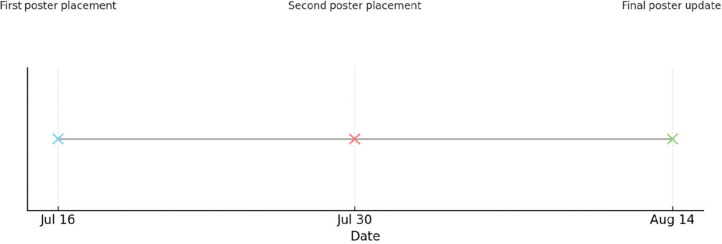
Timeline of poster placement.

This study employed a quasi-experimental design involving six buildings with three different treatments to evaluate the effects of informational and competition-based (with and without prize) nudges. The study took place over 4 weeks to observe both short-term and potentially sustained behavioural changes (shown by Fig. 1).

All the buildings in this study are located in London, and will be referred to as buildings A, B, C, D, E and F for ethical considerations. By focusing on properties within the same geographical and climatic region, the study controls for variations in energy usage that could be caused by extreme temperature differences, which are known to significantly impact heating and cooling demands [42]. In addition to temperature, the length of day and night plays a crucial role in energy usage, especially regarding lighting. London’s day length varies throughout the year, but all the buildings in this study experience these variations in the same way, thus eliminating a potential source of bias. Research by Maachi et al. [43] highlights the impact that natural light availability can have on energy consumption in buildings, particularly in residential settings where artificial lighting is a major energy consumer. Thus, by ensuring that all buildings were subject to the same climate and daylight patterns, this study was able to avoid extreme situations that could cause significant increases or decreases in energy usage among these buildings, as there were no extreme weather events happening in London during the treatment period. The six buildings were assigned to four groups based on their geographical proximity, with nearby buildings allocated to the same group to minimise potential cross-group contamination and ensure consistency in the environmental context [44]. The groups were as follows:

Control Group (Building D): No intervention. This group served as a baseline to measure the natural variations in energy consumption without any external influence.

Information-only Group (Building F): This group involved one building receiving biweekly feedback about their energy usage compared to their past consumption levels.

Competition Without Prizes Group (Building C and Building E): Students in two buildings competed to reduce their energy consumption. They were informed about the competition and provided with updates on their building’s performance relative to the other building biweekly, but no prizes were awarded.

Competition With Prizes Group (Building A and Building B): Similar to the competition without prizes, but the winning building received a tangible reward (a bottomless pizza night provided by the student accommodation provider). This group tested the added motivational effect of extrinsic rewards.

The competition between the buildings was based on the percentage reduction in energy consumption relative to a baseline that was uniquely defined for each building. This baseline was established by calculating the average daily consumption using historical data specific to each building. For example, the baseline consumption for Building A in July was determined by calculating the average daily consumption from the corresponding months in the years 2022 and 2023. This approach ensured that the baseline was reflective of the building’s typical energy usage patterns, allowing for a fair and accurate comparison of the percentage reductions achieved during the competition.

Although occasional heatwaves have drawn public attention in recent years, the average temperatures in London during July and August remained relatively stable between 2022 and 2024, indicating minimal climatic interference with the experimental conditions of this study. In 2022, while London experienced a record-breaking peak temperature of 40.2 °C, the overall average for July–August stayed around 21 °C, consistent with long-term seasonal norms [45,46]. Similarly, in 2023, despite being the eighth warmest summer on record in the UK overall, London’s daily averages during the same period remained within a narrow range of 20–22 °C, showing no extreme deviation from typical summer temperatures [47]. The summer of 2024 also exhibited this trend: although there were a few hotter days in late July (peaking at 32 °C), the average temperatures across the 2 months settled again at around 21–22 °C, with a slightly cooler August [48,49]. These observations suggest that, over this 3-year span, average summer temperatures in London were largely consistent, and therefore are unlikely to have significantly influenced energy consumption behaviour or confounded the study’s intervention effects in student accommodation.

### Participants

The study involved six student accommodation. All tenants who lived in these accommodation were full-time university students from different academic disciplines, nations, and years of study.

### Feedback

The posters were updated biweekly, a total of three times including the initial placement. This study placed all posters in the high-traffic area, the reception area/common room, where everyone would notice them each time they left or returned to make sure the posters were noticed by the students frequently. See Appendix A and B.

### Data collection

Energy consumption data and demographic data were provided by the student accommodation provider. Energy consumption data was collected using smart meters installed in each accommodation unit. These meters provided real-time data on electricity and heating usage, which was recorded on a daily basis throughout the study period. The data were aggregated to the building level to conduct the analysis. In total, the energy consumption data from 1 January 2022 to 29 August 2024 were provided by the student accommodation provider for each building.

## Results

### Descriptive analysis

As shown by Table 1 below, the six buildings have an average of 589 beds, ranging from a minimum of 370 beds (Building C) to a maximum of 707 beds (Building E). Additionally, the standard deviation (SD) of 153.7 indicates that a moderate spread in the number of beds across buildings. The mean gross internal floor area (ft^2^) is 91,007 ft^2^, ranging from a minimum of 78,033 ft^2^ (Building C) to a maximum of 252,459 ft^2^ (Building B). This shows that the sample represents a relatively large size scope from medium size buildings to large size buildings. Lastly, analysis of the students’ nationality shows that students from China represent the largest group, making up 41.6% of the population across the accommodation. UK students represent 16.6%, followed by the rest of the world (RoW) group at 22.4%, India at 12.0%, and the EU at 7.8%. More than half of the students are from China in two of the student accommodation: Building A (63%) and Building E (58%).

**Table 1.** Basic demographic information about the buildings

Asset name	City	Operational bed mix	Gross internal floor area (ft^2^)	UK	China	India	EU	RoW
Building A	London	436	177,514.506	7%	63%	6%	5%	19%
Building B	London	704	252,458.856	5%	38%	14%	13%	31%
Building C	London	370	78,033.403	49%	10%	17%	8%	17%
Building D	London	729	223,726.511	NA	NA	NA	NA	NA
Building E	London	707	233,654.148	12%	58%	8%	5%	17%
Building F	London	588	180,657.271	10%	39%	15%	8%	28%

The basic energy usage information for the buildings is shown in Table 2 below. Building C consumed the least amount of energy among all buildings, which is consistent with the fact that it has the least number of beds and the smallest gross internal floor area. In 2022, it had an average consumption of approximately 1806 kWh, with relatively moderate variability (SD: 484.07 kWh), ranging from 956 kWh to a maximum of 3077 kWh. In 2023, the average energy consumption increased to around 1946 kWh, and the SD also rose to 561.75 kWh, indicating greater fluctuation, with a peak of consumption at 3082 kWh. In 2024, although data is only available for part of the year (242 days), the average consumption increased further to 1976 kWh, with higher variability (SD: 597.50 kWh), and a maximum consumption of 3315 kWh.

**Table 2.** Basic energy usage information about the buildings

Building	2022 Avg (kWh)	2022 SD (kWh)	2022 Min (kWh)	2022 Max (kWh)	2023 Avg (kWh)	2023 SD (kWh)	2023 Max (kWh)	2024 Avg (kWh)	2024 SD (kWh)	2024 Max (kWh)
Building C	1806	484.07	956	3077	1946	561.75	3082	1976	597.50	3315
Building F	4599	1210.24	2817	7898	4277	1240.99	7182	4273	803.00	6761
Building B	3459	352.00	2483	3699	3884	546.73	4126	3936	446.14	4306
Building E	4443	1879.40	118	5967	5056	2251.00	6707	5115	2167.12	6876
Building A	3375	740.00	1988	3928	3092	811.00	3669	2847	863.50	3475
Building D	2948	407.40	2028	3649	2806	451.20	3694	2633	402.00	3273

Building F consistently showed the second highest energy consumption among all the buildings. This is worth noting as it was neither in the top three largest buildings nor did it contain the top three most number of beds. Thus, there must be some reasons causing this issue. It could be associated with its building design. For instance, the heating, ventilation, and air conditioning systems could be inefficient [50], and its orientation and window-to-wall ratios could also lead to high energy consumption [51,52]. In 2022, the average consumption was 4599 kWh with a substantial variability (SD: 1210.24 kWh), indicating large fluctuations, ranging from 2817 kWh to a maximum of 7898 kWh. In 2023, the average dropped slightly to 4277 kWh, but the variability remained high (SD: 1240.99 kWh), with a peak at 7182 kWh. These high figures reflect Building F’s energy-intensive nature. The huge fluctuations indicate this building is subject to huge seasonal occupancy level changes, as it experiences a significant decrease in energy consumption around May each year and then gradually rises back to high levels from around October. In 2024, the mean consumption was similar to 2023, which was 4273 kWh, with a much lower SD (803 kWh) than the past 2 years, showing a steadier usage of electricity ranging from 3210 kWh to 6761 kWh.

Energy consumption in Building B showed a consistent increase in energy usage. In 2022 it had an averaged consumption of 3459 kWh, with relatively low variability (SD: 352 kWh), ranging from 2483 kWh to 3699 kWh. In 2023, the average increased to 3884 kWh, maintaining similar variability (SD: 546.73 kWh), with a peak of 4126 kWh. In 2024, the average increased further to 3936 kWh, with slightly less fluctuation (SD: 446.14 kWh), and a peak of 4306 kWh. This suggests a constant increase in consumption over time.

Energy consumption at Building E remained the highest throughout the 3 years, which is reasonable as it was the largest building in terms of bed numbers and was second largest in terms of the gross internal floor area. In 2022, the average consumption was 4443 kWh, with extremely high variability (SD: 1879.4 kWh), ranging from 118 kWh to 5967 kWh. In 2023, the average increased to 5056 kWh with greater fluctuations (SD: 2251 kWh) and a peak of 6707 kWh. For 2024, the average consumption remained high at 5115 kWh, with a similar level of fluctuation (SD: 2167.12 kWh) and a peak of 6876 kWh. Such high fluctuation indicates that there must have been huge fluctuation in the occupancy pattern in this building throughout the years.

For Building A, the average consumption was 3375 kWh with a relatively high variability (SD: 740 kWh) in 2022, ranging from 1988 kWh to 3928 kWh. In 2023, the average decreased slightly to 3092 kWh, with similar variability (SD: 811 kWh) and a peak of 3669 kWh. By 2024, the average consumption further decreased to 2847 kWh with greater fluctuations (SD: 863.5 kWh), and a peak of 3475 kWh, indicating a slightly downward trend in energy use throughout the years.

Energy consumption at Building D also experienced a decline across the years. In 2022, the average was 2948 kWh, with lower variability (SD: 407.4 kWh), ranging from 2028 kWh to 3649 kWh. In 2023, the average consumption decreased slightly to 2806 kWh, with slightly higher variability (SD: 451.2 kWh) and a peak of 3694 kWh. In 2024, the average remained stable at 2633 kWh, with similar fluctuations (SD: 402 kWh) and a peak of 3273 kWh, indicating consistent energy consumption over time.

**Figure 2:**
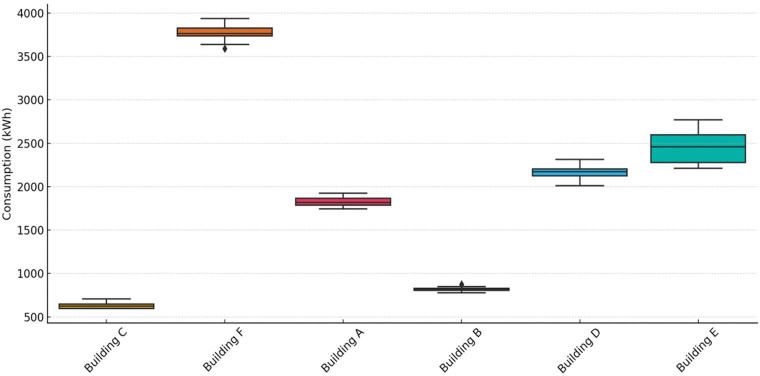
Box-plot for energy consumption during experimenter period (16 July – 13 August 2024).

Figure 2 presents a box-plot showing the distribution of daily electricity consumption across the six buildings during the intervention period from 16 July to 13 August 2024. Among these, Building C exhibited the lowest average consumption, which aligned with its smaller size and lower occupancy, recording a mean of 641.7 kWh and a relatively narrow interquartile range (IQR), indicating consistent daily usage. In contrast, Building F and Building E demonstrated the highest consumption levels, with means of 3765.4 kWh and 2460.9 kWh, respectively. Notably, Building F showed substantial variability with a wide IQR and several outliers, suggesting fluctuations possibly due to irregular occupancy or inefficient building systems [50,51]. Buildings A and B presented moderate average consumption values, around 1866.3 kWh and 825.3 kWh respectively, with relatively consistent usage patterns. Lastly, Building D demonstrated an intermediate profile, averaging 2187.7 kWh daily. Overall, this provided an important contextual baseline prior to the analysis of intervention effects.

### Difference-in-differences (DiD) analysis

To start with, a DiD model is used to analyse the effectiveness of different nudges compared to the Control Group. The data included in the DiD analysis are 16 July to 13 August’s data in 2022, 2023 and 2024, which is the treatment period. By only including this period, this analysis controls for any seasonal effects on energy consumption that would affect the results. Based on Abadie and Cattaneo [53], the specific model is summarised as:

$$\begin{array}{l} \text{Consumption}=\alpha +\beta 1(\text{treatment\_info})+\beta 2(\text{treatment\_comp\_prize})+\beta 3(\text{treatment\_comp\_no\_prize})\\ \;\;\;\;\;\;+\beta 4(\text{post\_treatment})+\beta 5(\text{treatment\_info\_post})+\beta 6(\text{treatment\_comp\_prize\_post})\\ \;\;\;\;\;\;+\beta 7(\text{treatment\_comp\_no\_prize\_post})+\varepsilon \end{array}$$

The dependent variable, Consumption, represents the daily energy consumption in kWh for each building. The model includes several key explanatory variables. First, treatment_info is a binary variable that equals 1 if a building is part of the Information-only treatment group and is 0 otherwise. Similarly, treatment_comp_prize indicates whether a building is part of the Competition With Prizes treatment group, while treatment_comp_no_prize captures the buildings in the Competition Without Prizes group. These variables allow for comparisons between the Control Group and each treatment group in the pre-treatment period, identifying any baseline differences in energy consumption. The variable post_treatment is a binary indicator that equals 1 for observations in the post-treatment period (after the interventions were introduced) and 0 for the pre-treatment period. This variable measures the overall shift in energy consumption across all groups following the implementation of the interventions. To capture the specific effect of the interventions on each treatment group, interaction terms were introduced: treatment_info_post, treatment_comp_prize_post and treatment_comp_no_prize_post. These interaction terms measure the additional effect of the treatment on energy consumption for each respective group during the post-treatment period. In other words, these coefficients reflect how much the energy consumption of the treated groups changes relative to the Control Group after the intervention. Robust standard errors were used in the model to ensure that the coefficient estimates are reliable.

**Figure 3:**
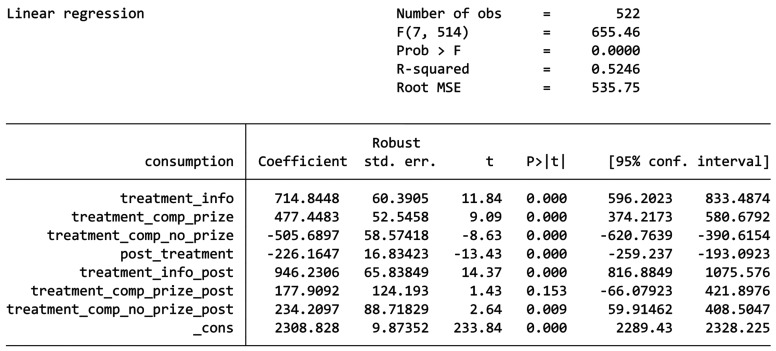
DiD results.

The results for the DiD analysis is shown by the table in Fig. 3. In the pre-treatment period, buildings in the Information-only group consumed 714.84 kWh more on average compared to the Control Group, buildings in the Competition Without Prizes group consumed 477.45 kWh more on average compared to the Control Group, and buildings in the Competition With Prizes group consumed 505.69 kWh less on average compared to the Control Group. All these are statistically significant (*p* < 0.001), indicating that before the treatment, the Information-only group and the Competition Without Prizes had significantly higher energy consumption than the Control Group, while the Competition Without Prizes group have significantly less. The Information-only group experienced an increase of 946.23 kWh in energy consumption during the post-treatment period relative to the Control Group, which is even higher than its difference to the Control Group before the treatment. This effect is statistically significant, indicating that the information nudge led to a significant rise in energy consumption instead of a decrease. The Competition With Prizes group experienced an increase of 177.9 kWh in energy consumption during the post-treatment period compared to the Control Group. Moreover, this effect is statistically insignificant suggesting that we cannot say the competition-based nudge has any effect in changing students’ energy consumption. Lastly, the Competition Without Prizes group saw an increase of 234.2 kWh in energy consumption during the post-treatment period relative to the Control Group. This is statistically significant and suggests that the competition without a prize led to an unexpected increase in energy consumption such as in the Information-only group.

**Figure 4:**
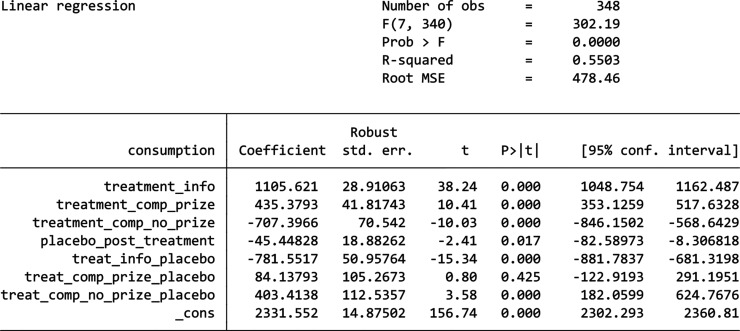
Robustness check results.

**Figure 5:**
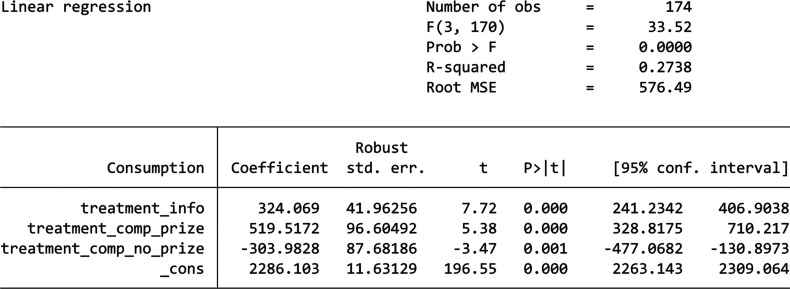
Falsification test results.

From the above DiD analysis, we can see that all three nudges failed to reduce energy consumption, which is not consistent with the previous studies’ results. Thus, a robustness analysis was done to check whether this DiD model is robust and reasonable. We first used a placebo test which pretends that the treatment happened in 2023 (instead of 2024) and analyse the data for 2022 and 2023 as if 2023 were the post-treatment period. As no intervention actually occurred in 2023, we expect no significant treatment effect in this placebo test. However, the coefficient for both the Information-only group (*p*-value = 0.0000) and the Competition Without Prizes group (*p*-value = 0.0000) are statistically significant, which indicates that external factors are affecting these two groups making them different from the Control Group. The results are shown by the table in Fig. 4. Then, we used another DiD model on 2023 alone as a falsification test, where we used only 2023 data and ran the DiD model with no actual ‘treatment’ period to test if any treatment effect appears when there should not be one. The results (the table in Fig. 5) showed an even worse case than the robustness check as all three treatment groups show a significantly different energy consumption compared to the Control Group (with all *p*-value ≤ 0.001). From the descriptive analysis we can also see that the Control Group exhibits a constant decrease in energy usage from 2022 to 2024 even without any treatment, while most of the other buildings exhibit an increase in usage, which shows that reduction in energy usage was not a natural trend among the buildings in London in these years. Therefore, something likely occurred in the control building that contributed to the observed reduction in energy usage. For example, improvements in the building’s design or the installation of more energy-efficient appliances may have gradually enhanced the overall energy efficiency of the building. Thus, it would be inappropriate to use a DiD model to compare these buildings simultaneously in this case.

From the descriptive analysis, we could also notice that different buildings exhibit unique characteristics, such as variations in occupancy rates, which is affecting the results. For instance, some buildings may experience a naturally significant drop in energy usage during the treatment period as students studying in schools in that region have holidays during July and will vacate during that time, while others may maintain a more stable population or even an increase in tenancy throughout the summer as there are summer schools in that region which would cause new tenants to move in. This variation in usage patterns can lead to substantial differences in energy consumption when compared between different buildings for a certain time period, independent of any interventions.

To ensure a more equitable and accurate comparison, this study would then adopt a longitudinal approach, focusing on the same building across multiple years for the same period of time (the treatment period). Comparing each building longitudinally offers a within-subject design, which is advantageous because it controls for fixed characteristics (such as size, layout, and tenancy patterns) and focuses on changes in energy consumption over time under consistent conditions. This helps isolate the effects of time-dependent variables, allowing researchers to better understand how interventions influence outcomes. Additionally, longitudinal comparisons are particularly well-suited to this study’s quasi-experimental design. Campbell and Stanley [54] emphasise that in quasi-experiments where randomisation is not feasible, tracking the same unit over time provides a clearer picture of how specific interventions or time-dependent factors impact the results. In this case, analysing consumption across three consecutive years allows for a more reliable identification of trends in energy usage, as it mitigates the confounding effects caused by inter-building variability.

Therefore, in the following section, this study first uses a multi-line time series plot to visualise the change in consumption between different years for each building and then tests the statistical significance of the difference between years using the t-test.

### Multi-line time series plot and t-test

#### Building C

**Figure 6:**
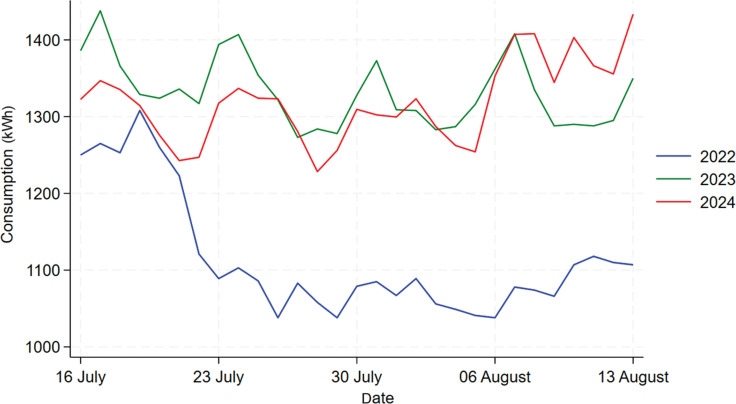
Comparison of daily electricity usage for Building C during treatment period across 2022, 2023 and 2024.

Figure 6 shows the time series plot comparing the energy consumption for Building C for the same period of 16 July to 13 August in 2022, 2023, and 2024. We can see from the graph that the daily consumption in 2022 shows a significantly different trend than the other 2 years, which makes it inappropriate to include 2022’s data in the analysis. For instance, there might be some large changes that happened to the building in 2022 due to post-pandemic policies. But the data for 2023 and 2024 exhibits a similar trend so we will focus on the analysis between these 2 years.

**Figure 7:**
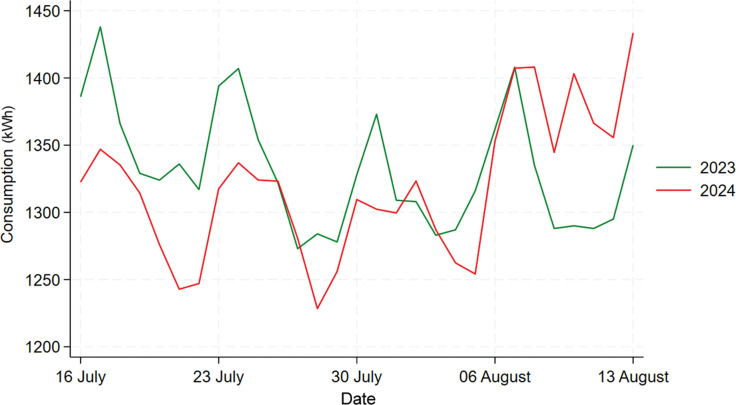
Comparison of daily electricity usage for Building C during treatment period between 2023 and 2024.

**Figure 8:**
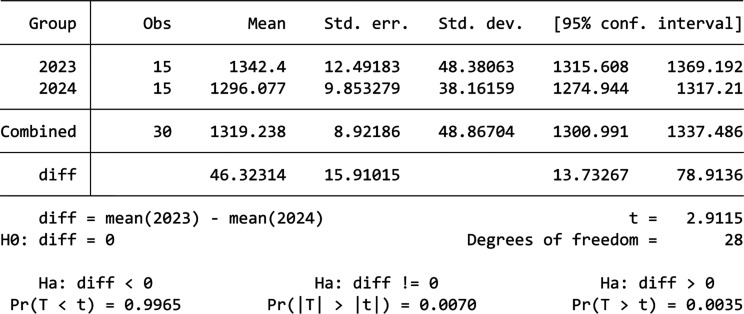
T-test result for Building C.

Figure 7 shows the comparison between 2023 and 2024’s data for Building C. We can see that, overall, there was no significant decrease in energy consumption in 2024 compared to 2023, but in the first 2 weeks there was an obvious reduction in consumption. To further find out whether the difference between these 2 years are statistically significant, a t-test would be performed. Firstly, the normality of 2023 and 2024’s data are checked using the Shapiro–Wilk test, which shows that which shows that both data are normally distributed with a *p*-value of 0.07099 and 0.6948. Then, the t-test results shows that the mean daily electricity consumption for 2023 is 1332 kWh, and the mean for 2024 is 1320 kWh, which did not show a significant drop in consumption. The *p*-value of 0.3993 further shows that this decrease in electricity consumption is not statistically significant. The t-test results for the first 2 weeks are shown by the table in Fig. 4. From the table in Fig. 8, we can see that the mean values for 2023 and 2024 are 1342.4 kWh and 1296 kWh, not a huge difference but given the fact that, overall, Building C’s mean daily energy usage in 2024 has increased when compared to 2023, shown by descriptive analysis, this decrease is also worth noting. Moreover, this decrease is statistically significant (*p*-value = 0.007), which means that the information nudge worked for the first 2 weeks in Building C.

#### Building F

**Figure 9:**
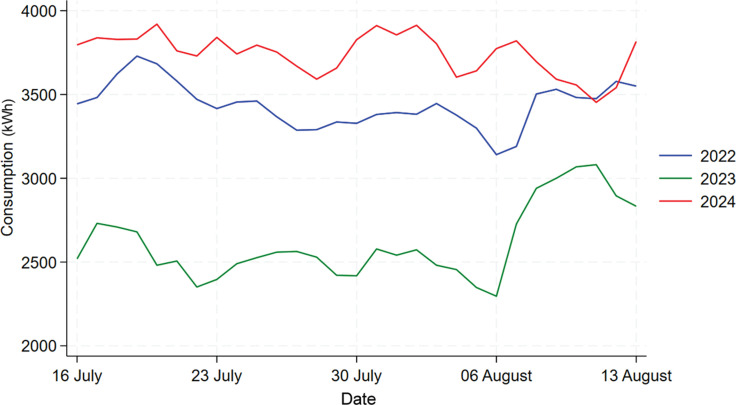
Comparison of daily electricity usage for Building F during treatment period across 2022, 2023 and 2024.

Figure 9 shows the time series plot comparing the energy consumption for Building F in 2022, 2023 and 2024. We can see from the graph that 2023’s daily consumption is much lower than that in 2022 and 2024, which means there might be some external factors affecting 2023’s consumption such as refurbishment of some rooms, thus it would be inappropriate to include 2023’s data in the analysis. The data for 2022 and 2024 exhibits a similar shape so we will focus on the analysis between these 2 years.

**Figure 10:**
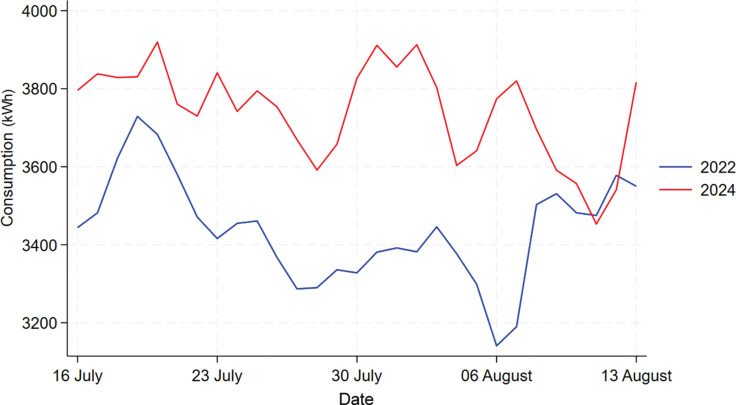
Comparison of daily electricity usage for Building F during treatment period between 2022 and 2024.

**Figure 11:**
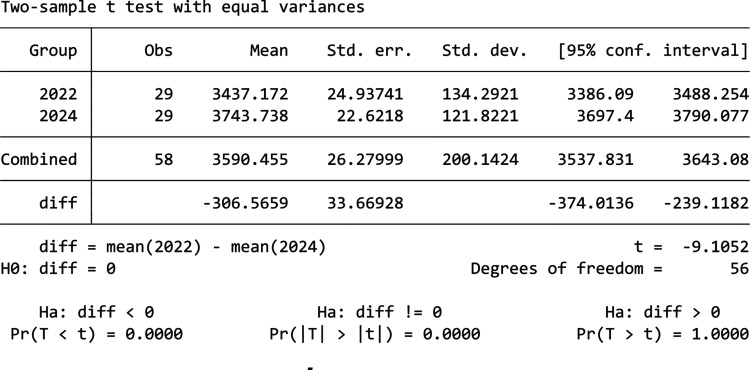
T-test result for Building F.

Figure 10 shows the comparison between 2022 and 2024’s data for Building F. From it we can see that there was actually an increase in energy usage in 2024 when compared to 2022 instead of a decrease. The normality of 2023 and 2024’s data are checked using the Shapiro–Wilk test, which shows that both data are normally distributed with a *p*-value of 0.989 and 0.094. Thus, a t-test is used shown by the table in Fig. 11, the result shows that mean consumption in 2022 and 2024 are 3437.1 kWh and 3743.7 kWh, and this increase in mean energy usage is statistically significant with a *p*-value of 0.000. Thus, this result shows that the information nudge is ineffective in reducing energy usage in this building, instead it increased the energy consumption.

#### Building B

**Figure 12:**
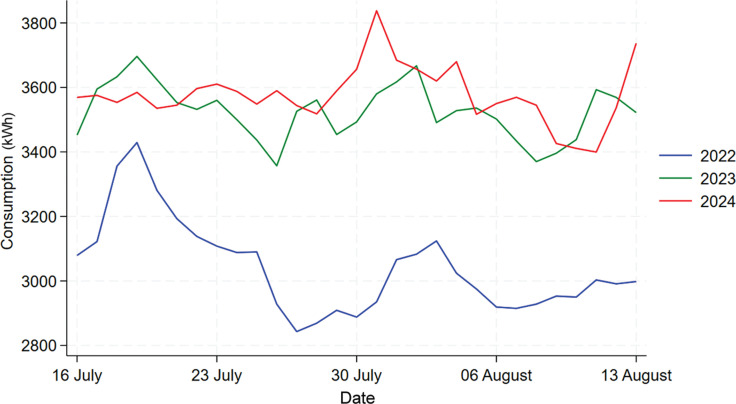
Comparison of daily electricity usage for Building B during treatment period across 2022, 2023 and 2024.

**Figure 13:**
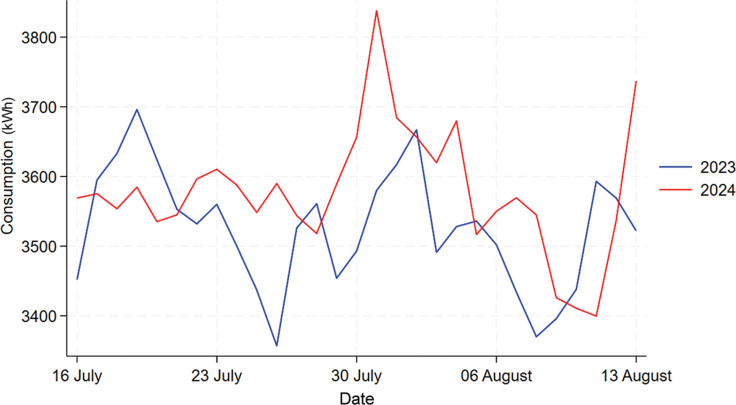
Comparison of daily electricity usage for Building B during treatment period between 2022 and 2024.

**Figure 14:**
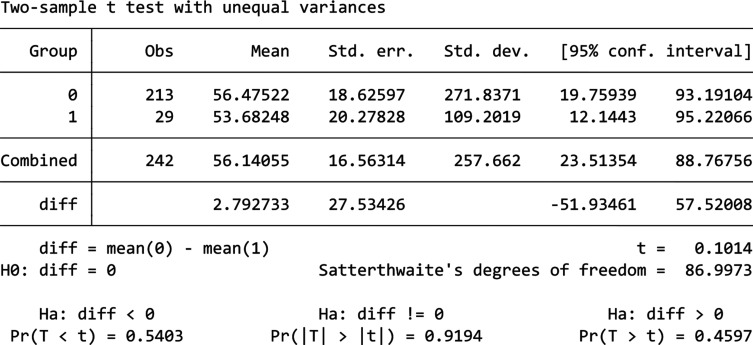
T-test result for Building B.

From Fig. 12, we can see that 2022’s daily consumption shows a much different trend than the other 2 years and therefore we would drop it from the analysis as before. The comparison between 2023 and 2024 is shown by Fig. 13, from which we can see that there is a slightly higher energy consumption in 2024 compared to 2023 during the treatment period. As the descriptive analysis also shows an increase in mean consumption in 2024 overall when compared to 2023, we would first compare the percentage increase in the mean consumption for the whole year to the percentage increase during the period in question. Overall, in 2024 Building B has experienced a 13.4% increase in mean energy consumption, and for the treatment period the percentage increase is 15% where we cannot see a significant difference between these two. To further analyse the nudge’s effect, a 1-year data set was calculated using 2024’s data minus 2023’s data for each day, then this 1-year consumption difference data was divided into two separate data sets, one containing only the treatment period while the other contained the rest of the days. Then, a Welch’s t-test was used to test whether the difference between these two datasets is significant, the results (the table in Fig. 14) show that the difference is statistically insignificant (*p*-value = 0.9194). Welch’s t-tests are used to test the data which do not have the same variance, the two separated datasets have unequal variance shown by the Levene’s test. Thus, we cannot say there are any significant changes in daily electricity consumption for Building B in 2024 during the treatment period compared to 2023, which means the competition with prizes nudge is ineffective in this building.

#### Building A

**Figure 15:**
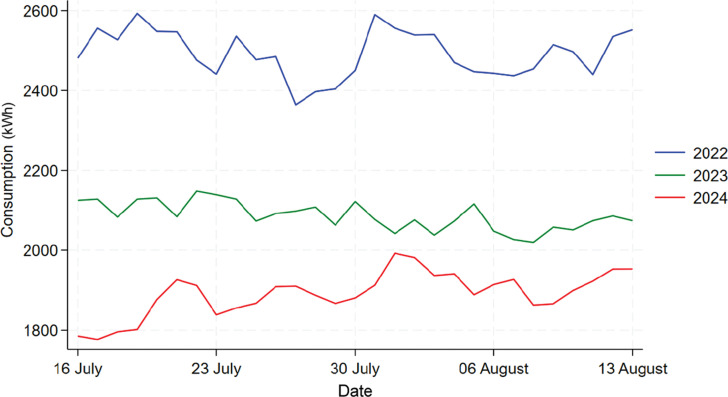
Comparison of daily electricity usage for Building A during treatment period between 2022, 2023 and 2024.

**Figure 16:**
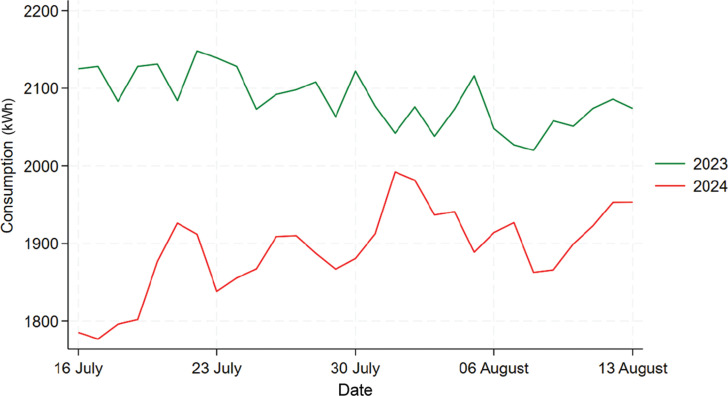
Comparison of daily electricity usage for Building A during treatment period between 2022 and 2024.

**Figure 17:**
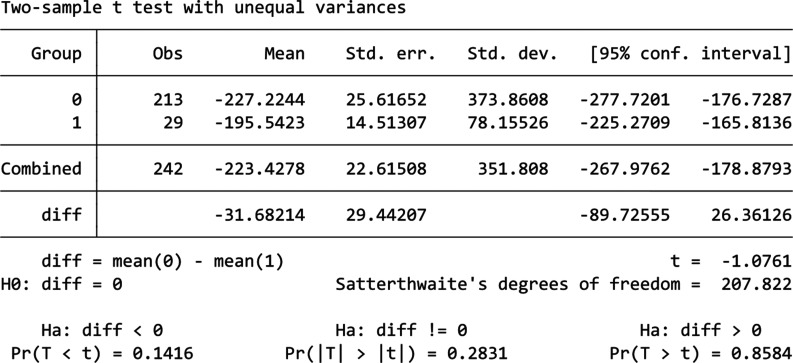
T-test result for Building A.

Figure 15 shows that the daily electricity consumption in 2022 needs to be excluded from the analysis as before. Figure 16, which only contains 2023 and 2024 data, shows a visually obvious decline in daily consumption throughout the treatment period in 2024. However, since overall 2024 also experiences a decrease in consumption shown by descriptive analysis, we need to compare these differences. Through calculation we can know that Building A in 2024 experienced an overall 7.94% decrease in the mean consumption, and for the treatment period the percentage decrease is 9.79%. Additionally, a Welch’s t-test such as the one used for Building B was used to test whether the consumption difference between the whole year and treatment period is statistically significant, the results (the table in Fig. 17) show that the difference is statistically insignificant (*p*-value = 0.2831), thus we cannot say the competition with prizes nudge has any significant effect in reducing electricity consumption in this building.

#### Building E

**Figure 18:**
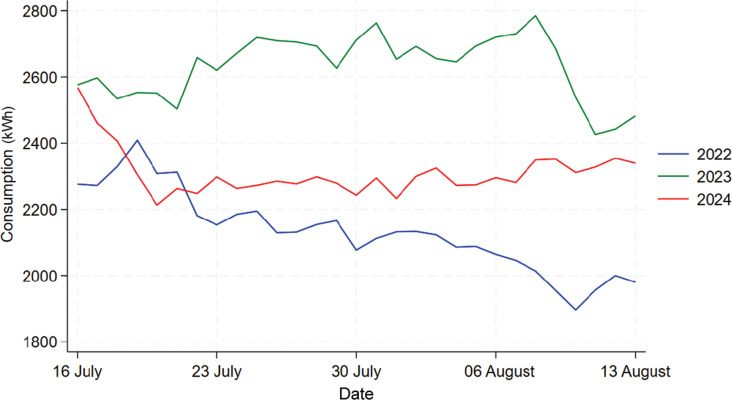
Comparison of daily electricity usage for Building E during treatment period between 2022 and 2024.

**Figure 19:**
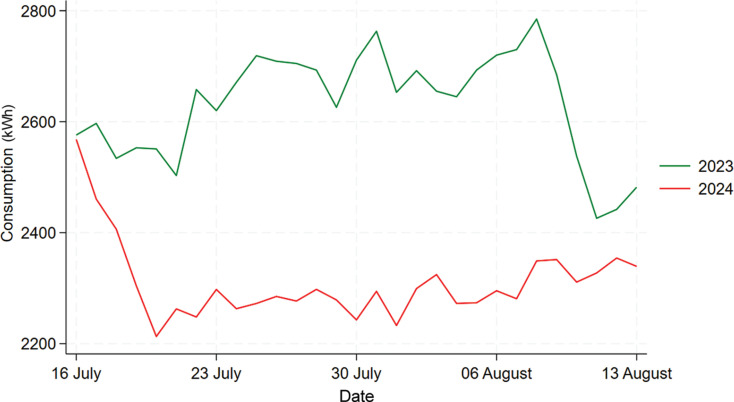
Comparison of daily electricity usage for Building E during treatment period between 2022 and 2024.

**Figure 20:**
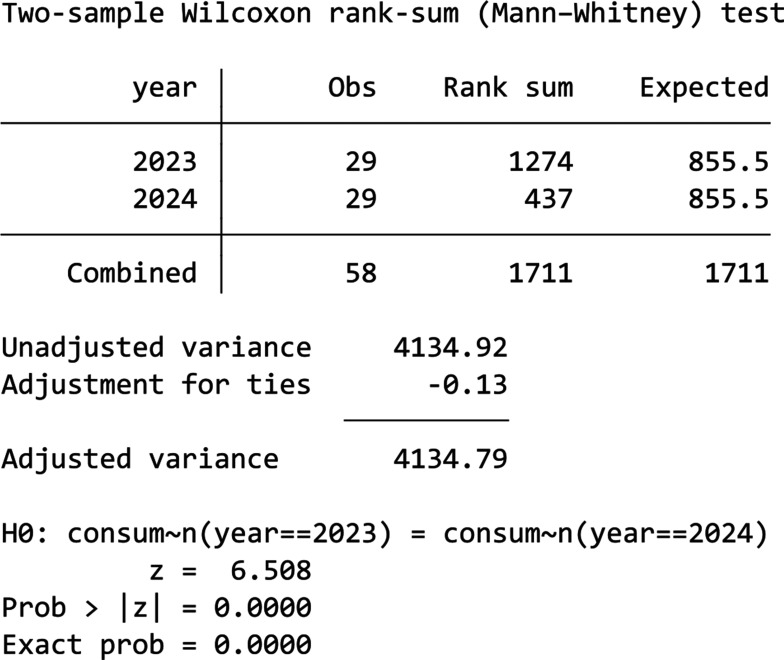
Wilcoxon rank-sum test for Building E.

From Fig. 18 we can see that there has been an unusual trend of decrease in energy usage since 18 July in 2022, thus we would drop 2022’s data from the analysis shown by Fig. 19. We can see from Fig. 19 that there is an obvious drop in consumption starting from the first day of treatment that lasted throughout the period in 2024. Additionally, from the descriptive analysis we know that 2024 actually has an increase in total mean daily consumption compared to 2023. Thus, this reduction in energy usage is worth noticing. However, since 2024’s data are not normally distributed when tested using the Shapiro–Wilk test (*p*-value < 0.05), a Mann–Whitney U test was used to test whether the difference between these 2 years is significant. The results (the table in Fig. 20) show that the difference is statistically significant (*p*-value = 0.0000). Thus, we could conclude that the competition without prizes nudge has worked effectively in reducing energy usage in this building.

## Discussion

Beginning with the DiD analysis, the results of the placebo and falsification tests suggest that the DiD model is not appropriate for this study due to external factors affecting the treatment and control groups differently. The assumption in a DiD model is that, absent the treatment, the control and treatment groups would follow parallel trends in energy consumption [53]. However, the observed differences in pre-treatment energy consumption and the significant effects found in the placebo test indicate that this assumption does not hold in this case. This violation of the parallel trends assumption means that the results of the DiD analysis should be interpreted with caution. This suggests that external factors have contributed hugely to the differences observed between the control and treatment groups in the post-treatment period by the DiD model. For instance, these factors could include changes in occupancy rates, seasonal effects or technological malfunctions in heating and cooling systems, all of which are known to impact energy consumption in buildings [55,56]. Specifically, when large numbers of students leave for holidays or extended breaks, the energy demand in residential buildings naturally declines, regardless of other interventions. Additionally, the Control Group exhibited a consistent decrease in energy usage from 2022 to 2024, despite receiving no interventions. This raises the possibility that there was something unique about the control building that contributed to this reduction, such as structural improvements, changes in occupancy or other energy-efficiency measures that were not implemented in the treatment buildings. Thus, for future studies which aim to investigate the effectiveness of different nudges using a DiD model in residential settings, the Control Group should not be a totally different building even within the same geographical location, as there are too many individual characteristics of different buildings which could affect the study results. The best situation would be a control group within each building (by floor).

The results from multi-line time series slot and t-tests show that rather than encouraging energy-saving behaviours, the informational nudge has increased the energy consumption in the building, which rejects our hypothesis 1. This is surprising given that prior studies, such as Fischer [26], have shown that feedback on energy consumption can be effective in reducing usage by making energy consumption visible and actionable for users. One potential reason why the informational nudge not only failed to reduce energy usage but may have led to an increase is the rebound effect or moral licensing. This occurs when individuals feel justified in using more energy after receiving feedback that they have saved energy compared to the past. The data shows that Building F actually consumed less energy in 2024 than in 2023, thus the comparison between the data students received 2024 and 2023 shows a reduction in energy usage in 2024. This might have inadvertently signalled to them that they were performing well, encouraging a sense of complacency. As a result, students may have felt they could afford to use more energy, believing they had already made sufficient progress. Moreover, informational nudges alone may fail to address deeper behavioural issues or may be too passive to provoke sustained change. That is, students at Building F may have simply ignored the information provided, or they may have been aware of their energy usage but lacked the motivation or personal accountability to make meaningful changes. Without a strong social or financial incentive to conserve energy, it is possible that students prioritised convenience or comfort (e.g., maintaining higher room temperatures or using appliances more frequently) over energy savings. Thus, future interventions should consider integrating behavioural reinforcement strategies (e.g., rewards, penalties or social norms) to enhance the effectiveness of informational nudges. Another reason for the failure of this information nudge could be the lack of customised data and energy-saving advice. A meta-analysis about information-based strategies by Delmas et al. [57] found that when it comes to encouraging conservation behaviour, techniques offering customised audits and advice work better than those that only offer historical, peer-comparison energy usage. Lastly, due to data and infrastructure restrictions from the student accommodation provider side, the study was unable to provide real-time feedback to students but only able to update the data biweekly, which could be one reason for the ineffectiveness of this nudge, as most studies that have previously shown significant results of information nudges have provided real-time data for students.

The results on the Competition Without Prizes group shows this nudge was effective in reducing energy consumption in Building E during the treatment period, and for the first 2 weeks in Building C, which supports hypothesis 2. The results from this group highlight the power of social comparison and intrinsic motivation as behavioural drivers. The competition likely created a sense of social accountability, where participants felt compelled to align with their peers in reducing energy consumption. This aligns with the social comparison theory, which posits that people tend to adjust their behaviour based on how they perceive themselves relative to others [30]. Moreover, the prolonged reduction in consumption throughout the treatment period in Building E suggests that the competition helped establish a new social norm around energy usage, where participants became more mindful of their consumption in a competitive, yet collaborative context. According to Cialdini et al. [58], social norms are particularly effective in promoting behavioural change when individuals feel that others around them are adopting similar behaviours. In competitive environments, individuals are motivated by a desire to outperform their peers and demonstrate socially desirable behaviours, such as reducing energy consumption [59]. It is interesting to note that, in this case, the lack of a material reward did not diminish the effectiveness of the nudge. This supports findings from studies such as Frey and Oberholzer-Gee [41], which suggest that in some contexts, extrinsic rewards (such as prizes) are not necessary to motivate behavioural change, and intrinsic motivations – such as the desire for recognition or achievement – can be equally powerful.

Another observation worth noting is that the nudge only worked for the first 2 weeks in Building C, which could be explained by two theories: the self-regulation theory and the cognitive load theory. The self-regulation theory posits that individuals have a limited capacity for self-regulation, which they draw upon to control impulses and maintain goal-directed behaviours [60]. However, as individuals continue to exert this self-control over time, they experience ‘ego depletion’, a state where their capacity to maintain the desired behaviour diminishes, leading to behavioural fatigue. Similarly, the cognitive load theory suggests that individuals have limited cognitive resources for processing information and making decisions (Sweller, [61]). When individuals are required to process frequent or complex information – as might be the case in interventions that involve continuous feedback on energy consumption – this can lead to cognitive overload, causing them to disengage from the task. In the case of the nudge in Building C, the initial novelty of the feedback or competition might have sparked interest, but over time, as the cognitive demand increased or the intervention became repetitive, participants may have lost motivation to actively engage. For future studies to counteract behavioural fatigue, interventions need to evolve over time to maintain participants’ interest and motivation. Strategies such as varying the feedback, introducing new incentives, or reducing the frequency of feedback to avoid cognitive overload while making the feedback more meaningful or personalised could increase its effectiveness over time.

The results for the Competition With Prizes group show that offering extrinsic rewards had no statistically significant effect on reducing energy consumption in the buildings during the intervention period, which rejects hypothesis 3. This outcome suggest that low-cost strategies which utilise intrinsic motivators may be more effective than those that provide extrinsic rewards to foster sustainable habits in student accommodation. Moreover, as competition without a prize shows an effective reduction in energy consumption, the ineffectiveness of prizes shown by this supports the crowding out theory introduced by Frey and Oberholzer-Gee [41], which states that in some cases price incentives could crowd out the effect of intrinsic motivation. Another possible reason for the lack of significant effect could be the social dynamics within the building. The social comparison theory [30] suggests that individuals are more likely to change their behaviour if they perceive that others in their peer group are also making similar changes. However, if the competitive element in the Competition With Prizes group was not well-publicised or if students did not feel a strong connection to the competition, the intervention might have failed to leverage the power of social norms effectively. Moreover, the specific type and value of the prizes offered may also have contributed to the intervention’s ineffectiveness. Research by Gneezy et al. [40] highlights that while extrinsic rewards can motivate behaviour, the magnitude of the reward often matters. If the prize offered in this competition was perceived as too small or not personally relevant to the participants, it may not have been enough to drive sustained behavioural change. Moreover, students may have felt that the effort required to win the prize (i.e., reducing energy consumption) was not worth the reward, leading to disengagement from the competition. Additionally, if the prize was a collective reward (e.g., a communal party or shared benefit), students may have felt that their individual actions would not significantly affect the outcome, leading to diffusion of responsibility according to Darley and Latané [62]. This study’s prize is a bottomless pizza night; thus, individuals may believe that their personal energy-saving efforts are unlikely to influence the group’s overall energy consumption, reducing their motivation to participate. Hence, further studies are needed to identify, firstly, whether it is really the case that prizes would crowd out effect on competition to decrease energy consumption in student accommodation settings; and secondly, whether more expensive prizes could overcome this crowd out effect and reduce more energy than competition without prizes.

This study also has several limitation. Firstly, due to restriction from the student accommodation provider’s side, the only available communication channel the author can use is posters. However, the use of posters may have been an ineffective and unengaging medium compared to more dynamic methods, such as energy dashboards or emails. Research on behavioural change interventions suggests that static communication tools such as posters often fail to capture and sustain attention, particularly in environments where individuals are constantly bombarded with competing stimuli [63]. More engaging communication channels, such as energy dashboards or email alerts, have been shown to be far more effective in fostering behavioural change. Energy dashboards provide immediate, real-time feedback on energy usage, allowing users to visualise the direct impact of their actions, which is crucial for reinforcing behavioural change [26,64]. Additionally, personalised emails can target individuals directly and offer tailored tips, reminders or updates, which are more likely to be noticed and acted upon. Research by Delmas et al. [57] found that personalised, timely feedback through digital means increased engagement and led to more significant reductions in energy consumption compared to generic information provided through passive channels such as posters. Secondly, the frequency and interactivity of communication play a key role in maintaining engagement. Emails and energy dashboards offer the possibility of ongoing interaction and frequent updates, which helps to sustain users’ interest and motivation over time. In contrast, posters are static and may become invisible to residents after initial exposure, leading to disengagement. Thus, by relying solely on posters, this study may have missed an opportunity to fully engage participants in the intervention. Thirdly, as the student accommodation provider was unable to supply the exact number of residents for each week, month, or year, this study could only use overall consumption data for comparisons. Ideally, per capita energy consumption data would have been more appropriate and would have yielded more accurate results. Moreover, due to restrictions from the student accommodation provider’s side, the author can only change the posters every 2 weeks, limiting the overall effectiveness of the nudges in driving sustained behavioural change. Lastly, the treatment period only lasted 4 weeks due to the long permission period required by the student accommodation provider, further studies could last longer to analyse the effect of the nudges over an extended time period or after they are finished, as studies have argued that these nudges could be less effective or ineffective in the long-run, or people would return to their original behaviour pattern after the nudges finish [65] (Bénabou and Tirole, 2003; [66]).

## Conclusions

To conclude, the results from this study reveal that among the three different nudges designed, only the competition without prize nudge worked effectively to reduce energy consumption. Due to the restrictions from the student accommodation provider’s side, this study has many limitations regarding communication channels and communication frequency which could have hugely affected the effectiveness of all the three nudges especially the information nudge. While the failure of the competition with prize nudge may also have been caused by the limits in communication, there is a growing body of studies showing that prizes may affect the effectiveness of the competition nudge adversely. Thus, more studies are needed to further examine whether prizes would have the opposite effect in the competition to reduce energy consumption in student accommodation. Lastly, this study shows that energy consumption in student accommodation is hugely affected by external factors and treatments usually only have a minor effect, thus when future researchers are designing research it is important to control for external factors.

The policy implications of this study are particularly relevant for universities, accommodation providers and policymakers seeking to promote sustainable behaviours in residential settings. The results suggest that competition-based nudges can be an effective, low-cost intervention to reduce energy consumption without the need for financial incentives. This is particularly beneficial for organisations with limited budgets, as traditional incentive programmes often require ongoing financial investment. However, for such interventions to be sustainable, competition structures must be carefully designed to prevent behavioural fatigue and maintain engagement over time. The failure of informational nudges highlights the need for more dynamic, real-time feedback systems, as passive informational approaches may not be sufficient to drive change. Policymakers should consider implementing digital feedback tools that provide personalised energy consumption insights, as studies have demonstrated their effectiveness in increasing conservation behaviours [64]. Additionally, this study suggests that offering extrinsic rewards does not necessarily enhance engagement and, in some cases, may even diminish the effectiveness of competition-based interventions. As a result, policymakers should prioritise intrinsic motivation strategies, such as social comparison, public recognition and gamification, rather than relying solely on financial incentives.

In conclusion, this study provides new evidence on the effectiveness of informational and competition-based nudges in student accommodation, highlighting the importance of intervention design, communication strategies and motivation dynamics. The findings reinforce the role of social norms and intrinsic motivation in driving behavioural change while underscoring the limitations of static informational nudges and extrinsic incentives. As climate change and energy conservation become increasingly urgent concerns, policymakers and accommodation providers must adopt behaviourally informed strategies that are engaging, cost-effective and scalable. While nudges offer a promising approach, their effectiveness ultimately depends on how they are structured, communicated and sustained over time. Future research should continue to refine these interventions, exploring long-term behavioural effects, optimal incentive structures and more personalised engagement methods to support sustainable energy consumption in residential communities.

## Data Availability

The datasets generated during and/or analysed during the current study are available from the corresponding author on reasonable request.

## Appendix

### Appendix A

#### Poster design

The posters are updated biweekly, a total of three times including the initial placement. To ensure clarity and effectiveness, this study designs the posters based on several well-established principles of cognitive psychology and design.

#### Cognitive load theory

Minimising cognitive load improves comprehension and retention by reducing extraneous effort in processing information 67. In this study, the posters are designed to deliver a concise message, avoiding excessive text or complex graphics. Instead, they provide a single, clear statement that can be understood at a glance, supporting the principle that simpler visual information enhances learning 68.

#### Visual hierarchy

A clear visual hierarchy ensures that key information is noticed first 69. This principle is applied in the poster design by using bold, large fonts for the main message and placing it at the top where it is most likely to be read first 70. Furthermore, Gestalt principles, particularly figure-ground distinction, aid in directing attention by making key elements visually stand out from their background 71.

#### Colour psychology

Colours influence perception and behaviour, making them a crucial element in poster design 72. Green, for instance, is associated with environmental awareness and safety, which aligns with the study’s goal of encouraging sustainable behaviours 73. The posters predominantly use green to subconsciously reinforce this message and encourage action.

#### Dual coding theory

The combination of textual and visual information enhances recall and understanding 74. By integrating relevant images alongside the text, the posters leverage dual coding to make the content more memorable and engaging 75. The visuals used directly relate to the behaviour being promoted, ensuring coherence between image and message.

#### Strategic placement and frequency

The location and frequency of exposure influence a poster’s effectiveness. To maximise visibility, all posters are placed in high-traffic areas, such as the reception area, ensuring that students see them regularly. Frequent exposure reinforces the message and increases the likelihood of behavioural change 72.
